# Reply to von Blanckenburg et al.: We provide a solution to the Neogene beryllium conundrum

**DOI:** 10.1073/pnas.2208945119

**Published:** 2022-08-08

**Authors:** Shilei Li, Steven L. Goldstein, Maureen E. Raymo

**Affiliations:** ^a^Key Laboratory of Surficial Geochemistry, Ministry of Education, School of Earth Sciences and Engineering, Nanjing University, Nanjing 210023, China;; ^b^Lamont-Doherty Earth Observatory, Columbia University, Palisades, NY 10964;; ^c^Department of Earth and Environmental Sciences, Columbia University, Palisades, NY 10964

Whether Neogene erosion and weathering increased is an important question with implications impacting our understanding of how Earth’s long-term carbon cycle works. We welcome the opportunity to respond to the concerns (*in italics*) raised by von Blanckenburg et al. (1) about our beryllium cycling model (2).

**Fig. 1.**
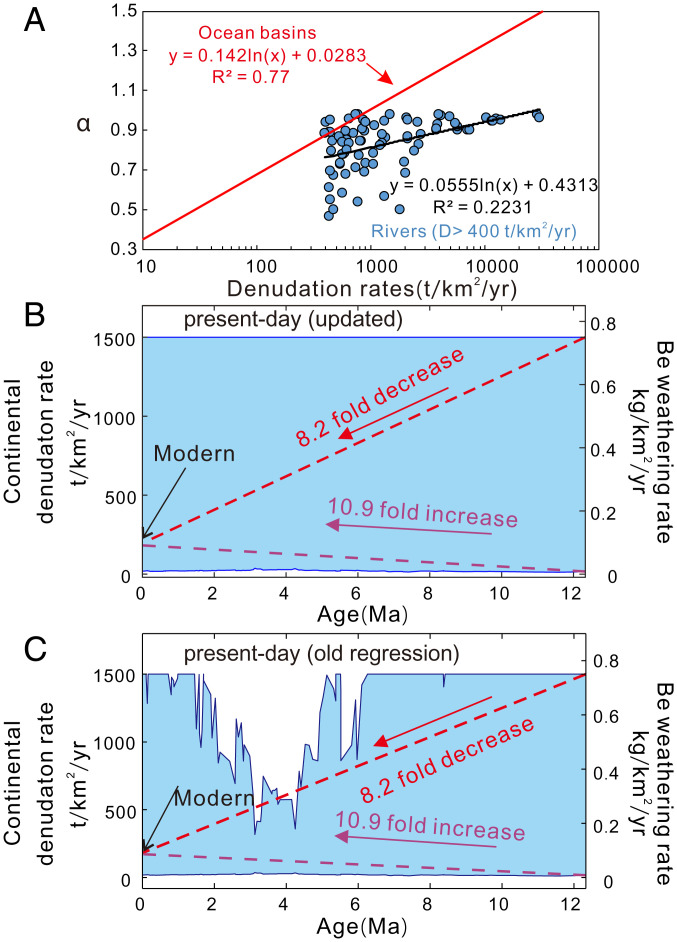
The Li et al. (2) model result changes by correcting the α value under high denudation rates. For the correction, when denudation rates are over 400 t*⋅*km^−2^*⋅*y^−1^, we use the global river regression (blue line in *A*, produced from data in ref. 9) instead of the ocean-basin regression (red line in *A*). (*B*) The modeled Cenozoic history of continental denudation and Be weathering with α corrected based on the “present-day” scenario of our model. (*C*) The modeling results under the “present-day” scenario originally presented in ref 2. It is clearly shown that correcting α results in minor changes in the modeling results. The updated modeling results are still consistent with an up to 11-fold increase in continental denudation during the past 12 My.

**Fig. 2.**
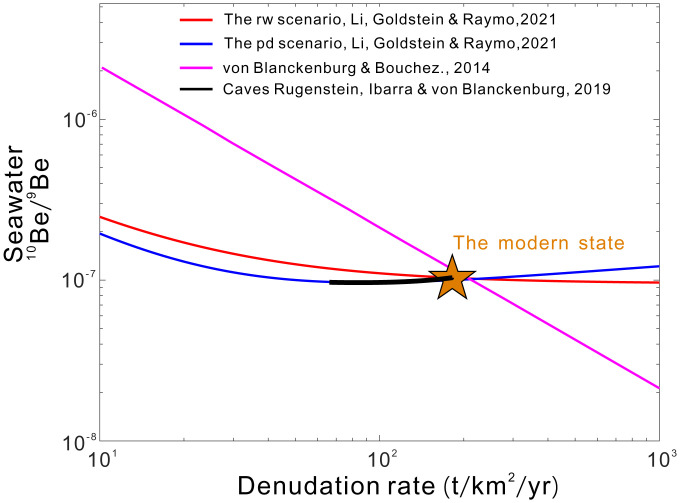
The relationships between seawater ^10^Be/^9^Be and denudation rates modeled by different studies. The red line and blue line represent the results produced by our model (2) under the “reworked-clay” and “present-day” scenarios, respectively. The purple line stands for the modeled relationship by ref. 3, which assumes that the fraction of Be entering the open ocean remains constant when denudation rates change. The black line represents the model results of another model by the von Blanckenburg group (8) based on both the seawater Be and Li isotope records.

1) *Equations 8* *and* *11 are dimensionally incorrect, impacting our conclusions; also, we assume linearity between ocean particle concentrations and physical erosion rates*. Equation 8 of ref. 2, which sets the initial particle concentration (particle flux per year/the volume they occupy in coastal oceans), should be F*t/V (where F, V, and t are the flux, volume, and time interval, respectively), rather than F/V. With this clarification, both equations are dimensionally correct, and since t = 1 y, the outcomes are unchanged.
2) *Our parameter “α”* (*physical erosion to denudation*) *cannot exceed 1 but does so when D > 1,000 t⋅km^−2^⋅y^−1^.* We concluded, based on the α-vs.-D relationship in ocean basins (figure S1 of ref. 2), that the Neogene record ^10^Be/^9^Be_seawater_ is consistent with a significant increase in global D from ∼17 to ∼182 t*⋅*km^−2^*⋅*y^−1^, which is far below 1,000 t*⋅*km^−2^*⋅*y^−1^. Using river data (where D can be >1,000 t*⋅*km^−2^*⋅*y^−1^), we have reevaluated the α-vs.-D relationship for rivers where D > 400 t*⋅*km^−2^*⋅*y^−1^. We find that α does not come close to exceeding 1 on Earth and that using the updated relationship still supports our conclusions (Fig. 1).
3) *The von Blanckenburg and Bouchez* (3) *model reproduces modern* ^10^Be/^9^Be_seawater_ *in shallow ocean water better than ours.* For the calculation of ^10^Be/^9^Be_seawater _in shallow ocean water, it is important to consider the Be flux advected from adjacent ocean basins; their model (3) does not, while our model does. Moreover, ours yields better matches between modeled and observed ^10^Be/^9^Be (figure 1 of ref. 2).
4) *We should consider the particle concentration effect* (*higher particle concentration leads to lower K_d_* [*adsorbed Be on suspended particles/dissolved Be*])*, even though it is an artifact* (*treating colloidal Be as dissolved Be*)*, because colloids help transport Be to the open ocean.* While organic colloids facilitate Be transport across estuaries, inorganic colloids and inorganic/organic colloid mixtures enhance scavenging (4). Moreover, higher erosion rates result in greater transportation of inorganic/organic colloids to estuaries (5), which favors Be scavenging and thus buffers ^10^Be/^9^Be_seawater_.
5) *Our model overlooks the boundary exchange* (*BE*) *Be flux.* The modern BE flux (135 t/y) (4) is only 26% of the open ocean Be flux (520 t/y, from ref. 2). Therefore, considering this would not significantly enhance the sensitivity of ^10^Be/^9^Be_seawater_ to denudation.
6) *Why do Be isotopes appear inconsistent with the other weathering proxies*? Unlike Be, scavenging has little impact on the riverine Li, Sr, and Os fluxes (6, 7), making seawater ^7^Li/^6^Li, ^87^Sr/^86^Sr, and ^187^Os/^186^Os sensitive monitors of continental denudation and weathering; if scavenging is not carefully considered, the constancy of ^10^Be/^9^Be_seawater_ appears inconsistent with these other records. We note that Rugenstein et al. (8) have recently acknowledged that Neogene denudation increased while ^10^Be/^9^Be_seawater_ remained constant (as our model predicts [Fig. 2]) and suggest the ^10^Be/^9^Be_seawater_ constancy indicates that the weathering flux was constant despite the increased denudation rate. In contrast, our results show that ^10^Be/^9^Be_seawater_ is insensitive to weathering flux. Our model (2), accounting for the effects of scavenging, provides an explanation why Neogene ^10^Be/^9^Be_seawater_ has remained constant and is thus consistent with the other proxies indicating accelerated denudation and weathering.
